# Medium-Sized Cities Facing the Demographic Challenge in Spain’s Low-Density Regions through Citizen Participation Projects

**DOI:** 10.3390/ijerph19095303

**Published:** 2022-04-27

**Authors:** María Ángeles Rodríguez-Domenech

**Affiliations:** Department of Geography, University of Castilla-La Mancha, 13001 Ciudad Real, Spain; mangeles.rodriguez@uclm.es

**Keywords:** medium-sized cities, demographic challenge, citizen participation, sustainable urban planning

## Abstract

The roles of medium-sized cities in processes of demographic challenge have taken many different paths. New forms of urban sprawl, deconcentrating processes, and the emergence of the diffuse city have marked a change in the relations that Spanish medium-sized cities have traditionally had with their most directly influenced territories. In line with the theoretical framework of the European next generation urban regeneration programme, the main aim of this paper is to propose a methodology to develop a project that fosters resilience strategies and the revitalization of local environments. This will also benefit the institutions that are involved in promoting it. The innovative methodology employed has been denominated the “We Propose!” project and has received several national acknowledgments. This is a strategically designed civic participation urban renewal project and has been subject to geographical analysis through field trips and in situ research. A case study into urban renewal strategies was carried out in Ciudad Real, which is a medium-sized city in Spain’s third largest region. It includes an evaluation of both the design and implementation of what could be considered a successful case of urban renewal carried out in the city. This urban development initiative was undertaken by the public administration, but it was designed and proposed by local citizens.

## 1. Introduction

The current debate is focused on the intricacies of the delimitations of the city, in which it is no longer able to utter of the dichotomy between “urban” and “rural” [1] due to the generalization of urban fact and its scattered growth. 

The urban sprawl has been examined in numerous occasions and diverse contexts. Whilst the majority of these studies have addressed major cities as well as metropolitan areas, recently these analyses have been further expanded seeing how (sub)urbanization development outside the traditional city limits also affects smaller cities [2]. This article pays attention to what has happened in city outskirts, with the complexity associated with the conceptualization of medium-sized cities and urban sprawl. Thus, only recently it has begun to analyze the dynamics in medium-sized cities’ outskirts as well as major metropolitan areas. 

Studies about the artificialization of the land in Spain, such as [3] or [4], throw light on the significance of the growing of so-called surroundings of central cities; that is to say, they distinguish between the center, corresponding to the center township (or, more importantly, the processes of urban sprawl in the surroundings) and the rest of municipalities that take part in the agglomeration. Hence, the main municipality would correspond with a vision of a more traditional city, whilst the surroundings would match up with the spaces of transition, of urban sprawl, as well as dissolution with the rural environment. In Spain, the greatest growth in artificial surface area is located, precisely, in surroundings of the city towards its periphery, at 60%.

The concepts of medium-sized or intermediary cities utilized in this paper will remain indifferent, based on the definition given by [5], who defines these cities as “urban settlement of medium-sized that perform urban functions such as intermediation amongst all centers of hierarchy levels” and, from this purely quantitative perspective in the European Union, settle limits between 50,000 and 500,000 inhabitants [6].

Our goal is to identify the different actions of the participatory citizen project of medium-sized cities in order to apply this project to their urban sprawl. We also aim to understand the local responses of cities and to identify the responses to the need to shift the city’s path. These and other issues can contribute to defining the role of medium-sized cities in the territorial context and in the face of the population challenge, as well as to looking more thoroughly at how to promote territorial solidarity in the rural sprawling area of medium-sized cities through the local urban renewal of medium-sized city centers. This study case about urban renewal strategies was carried out in Ciudad Real (75,045 inhabitants), a medium-sized city in the third largest region in Spain territorially, with 19,813 km^2^. This region, due to its characteristics and territorial distribution, can serve as an observatory of the role of medium-sized cities in Spain, which includes 102 municipalities, 80% of which are rural and semi-rural (less than 10,000 inhabitants). This distribution of population makes these medium-sized cities the principals and unifying hearts of the territory. Studies of how these cities work in their surroundings would favor the comprehension and the scope for such plans of action.

## 2. Background 

### 2.1. The Role of Medium-Sized Cities in the Light of the Demographic Challenge

In Spain, 81% of inhabitants reside in cities, 18% in big cities, and 17% in municipalities with fewer than 50,000 residents, of which the main core is more than 30 km in a medium-sized city. Therefore, the majority of the citizens (64%) reside in medium-sized cities or surroundings or “urban crowns”, which is a new concept from diffuse medium-sized cities that refers the spaces, including the municipalities that have their own town council and local government within this radius, with a radius of 30 km from each of the central cities [7] (p. 7). 

Nowadays, it is intelligible that urbanism together with spatial planning of the 21st century cannot seek to perpetuate past practices. This means that it is no longer possible to base on the unique economic growth principle as well as the territorial expansion without considering the effects of the quality of life, social development, or even the equilibrium of ecosystems [8] (p.26) and [9]. 

From European guidelines, medium-sized cities have been considered the urban category with better qualities for urban-territorial sustainability [10], which has fostered the augmentation of its protagonist of spatial planning, including development strategies, and in policy frameworks [11] (p.145). From the beginning of the implementation of Agenda 2030, the focus has been placed on the analysis of the roles of medium-sized cities beyond Goals 11, recognizing its significance for the attainment of the goals set. From different organizations it has been repeated that the battle for sustainable development will take place in cities, due to the fact that it is where the citizens are concentrated, where 80% of available resources are already consumed, and where more than 70% of global emissions are produced [12]. 

In Spain in recent decades, until the 2008 crisis, there has been an intense process of dispersed urbanization that has affected the entire Spanish territory with considerable growth in artificialization of the land [13]. In this urban process, urban intermediate areas have experienced the greatest relative increases, well above of those that present the main urban agglomerations [14]. It is in the municipalities located in the urban crowns of the medium-sized cities where the dynamics of city production has been more intense and accelerated since the beginning of the 21st century, converting territories traditionally considered rural in post-urban, with a growth rate greatly higher than in the respective cities that they depend on [15] (p. 301).

At present, it is utterly extended that the population encounters a diffuse city model, representing discontinuous, fragmented, low-intensity, and weakly cohered urban areas that are diluted in a suburban area and rururban territory [16], a city model that is more and more enlarged in Spain.

The medium-sized cities have been receptors of demographic effects originating near rural municipalities, which has generated positive dynamics in them from the point of view of both natural growth and migration. They have been areas that have demographically estructured the region, although there have been occasions of strengthening of the header having negative repercussions on the demographic decline of some of its surrounding rural municipalities [17]. Nonetheless, intermediary cities, situated between large metropolises and rural settlements, favor the dissemination of services, equipment, and knowledge to the territory, ensuring an adequate quality of life for its inhabitants and acting as activating centers in its surrounding [18]. In the Spanish case, these intermediary cities have been the center of a debate on their role in balance of territory as well as development promotion.

There exists a profound need to better comprehend the current dynamics of these cities, including applying strategies to convert them in specific resource centers that are able to disseminate knowledge and services that allow to favor the dynamization of rural areas’ surroundings, as signaled in some previous works as the one by [15,19,20,21,22]. Notwithstanding, the current situation and the recent dynamism of these cities should not be forgotten; due to the freeze on the construction of new dwellings, they have been left half-finished or unused, giving place to the urban landscapes of the crisis [23]. Thus, from a sustainable territorial development perspective, it has made landscapes for reflection of problematic solutions, with case studies still being scarce, so as to deepen in these new unfinished spaces where there is an urgency to search for resolutions that economically boost sustainable territorial evolution. 

The new forms of urban sprawl of deconcentrating and diffuse cities undergoes a change in the relations that the Spanish medium-sized cities have had traditionally with their most directly influenced territories. These mainly rural cores are in a difficult position where the loss of identity coexists with the unsustainability of this urban system of growth. Nonetheless, they have an effect of maintenance on its population as well as the creation of recently developed activities and services unrelated to the traditional rural world. 

### 2.2. The European Policies of Participatory Urban Regeneration Next Generation

Agendas, strategies, and legislation place the issue of urban regeneration in the centre of public policies, as it is considered a State affair that must be approached from a territorial cohesion perspective. Thus, focus is placed on the new economy that undoubtedly involves sustainability, digitalization, and service delivery of basic social services in all the points of the planet, mainly in those territories that are situated in a complicated situation of poverty including exclusion.

*United Nation International Agenda* [24] with 17 Sustainable Development Goals that target 169 integrated and indivisible comprising goals that envelop economic, social, and environmental spheres, with the final intent of eradicating poverty, protecting the planet, and guaranteeing that each and every one without distinction rejoice in peace and prosperity. 

*European Urban Agenda* [25] is an integrated and coordinated viewpoint towards the policies that have a potential impact in urban areas and contributes to a territorial cohesion. It opts for an integrated vision of sustainability together with posing objectives that ameliorate regulation, financing, and expertise.

*Post Covid “Next Generation EU” Recovery Funds* (2020) is a supplement to the UE regular budget that has been created in order to repair the immediate economic and social damages caused by COVID-19. Their intention is to counteract with investments as well as reforms the impact that the pandemic has had in the economies of the EU countries and to make both economy and employment more sustainable, resilient, and solidly prepared for future scenarios. Every seven years, the European Union equips with a budgetary framework that covers each period. In this latest one, which runs from 2021–2027, the total unprecedented import upgrades to 1.074 billion euros. With Next Generation EU, 750,000 million euros are added additionally, therefore, the total budget of the EU will approximate to almost double of the originally planned, gaining more than 1.8 billion euros.

*Spanish Urban Agenda 2030* (2019) is a strategic, non-regulatory document that promotes a new vision of Urbanism. It is composed of 10 objectives to achieve, adding a list of possible lines of actuation so as to be deployed for each actor who is willing to compromise with the Agenda. There are 10 Strategic Goals (SG):

SG1: To organize the territory and make a rational use of soil, conserving as well as safeguarding it. 

SG2: To circumvent urban dispersion together with revitalizing the existing city.

SG3: To avert and reduce the impacts of climate change along with ameliorating resilience.

SG4: To create resource management and favor a circular economy. 

SG5: To enhance proximity with sustainable mobility.

SG6: To nurture social cohesion including seeking equality.

SG7: To boost and vouch for the urban economy.

SG8: To guarantee access to housing

SG9: To introduce and stimulate digital innovation

SG10: To enhance the instruments of intervention together with governance. 

*Spain Can, Recovery Plan, Transformation and Resilience* (2020) is an elaborated document to embrace new mechanism that will accredit up to 140,000 million euros to Spain in transfers and credits in the period of 2021–2026, which will unite to the rest of tools foreseeing in the Pluriannual Financial Framework in regard to boosting investments together with reforms in priority areas at the European level. 

The recovery plan incorporates a consequential investment agenda as well as structural reforms that are interrelated and feed back to earn four transversal objectives: (1) Ecological Transition. Green Spain; (2) Digital Transition. Digital Spain; (3) Gender Equality. Spain Without Gender Gaps; and (4) Social and Territorial Cohesion. Cohesive and Inclusive Spain

*Integrated Territorial Investment Plan* (ITI) is a plan targeted at boosting new activities in specially depressed areas due to depopulation issues as well as socioeconomic decline. It delimits areas in each of the provinces of Castilla-La Mancha (2017) so that, after the appropriate diagnosis and backed by the European Union Regulation that articulates ITI as the tool enabled for the community regulation in support of integrated territorial actions that permits the combination of financing linked to diverse thematic objectives and to multiple programs bolstered by European Structural and Investment Funds as European Structural and Investment Funds (ESIF): European Regional Development Fund (ERDF), European Agricultural Fund for Rural Development (EAFRD), and European Social Fund (ESF), including a positive discrimination for being in this situation. 

### 2.3. Participatory Urban Regeneration Next Generation

From the mid-twentieth century to the present day, political authorities have been financing programmes to promote urban renewal, primarily in historic areas, but also in other types of neighbourhoods. Following Rubio-Huerbas and Ureña-Frances [26] (pp. 48–49), we can distinguish four key approaches to urban regeneration in the Western world, which are determined by the interests pursued.

The first wave of urban renewal, launched in the 1950s, was characterized by renovation (or reconstruction) projects, conducted by public administrations, which consisted of interventions based on resolving the problems of marginalised areas of cities through the replacement of substandard housing and the relocation of their inhabitants.

A second wave of urban regeneration was undertaken in the 1970s and 1980s and placed the emphasis on rehabilitation of both dwellings and public spaces. This saw the introduction of the concept of integrity as an essential element for achieving improvements of not only the physical environment, but also of its inhabitants, through the implementation of social action capable of involving civil society in these processes through what came to be known as “civic participation” [27].

The third wave, which began in the early 1980s, was one of regeneration and involved specific action within the city itself. It implied a number of alliances between public and private authorities, both at the individual level and with business agents.

The fourth wave of regeneration had its origin at the end of the 1990s and at the beginning of the 21st century. It emerged as part of a paradigm shift that brought with it a global housing crisis and that has focused on the revitalisation of neighbourhoods and even of whole cities and the promotion of inclusive participation and private-public alliances [28]. These actions have focused on physical spaces and on people, with cultural projects as the main protagonists and using them as catalysts for processes to revitalize degraded neighbourhoods and give them a new lease of life.

## 3. Study Area

### 3.1. Geographical Characterisation of the Study Area

**Figure 1:**
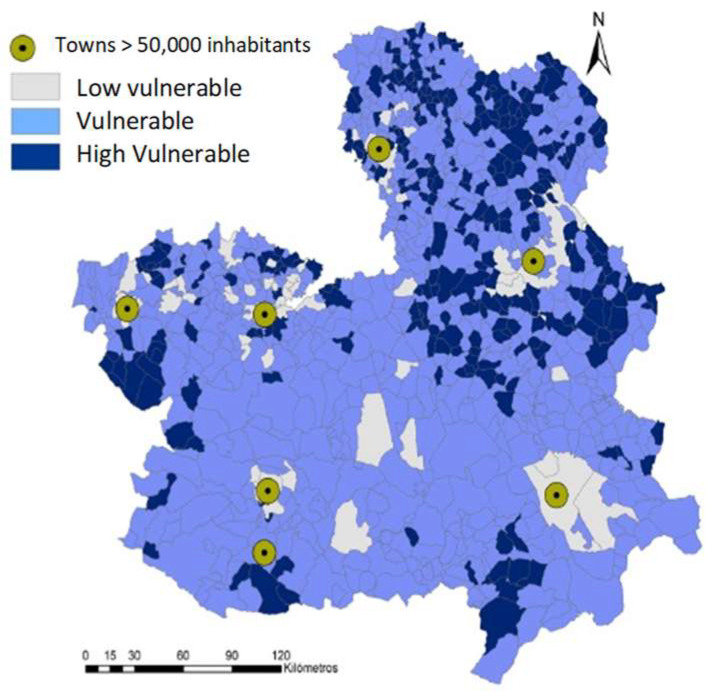
Vulnerability in Castilla-La Mancha, Source: [29] (p. 189).

Castilla-La Mancha is a pioneer region in Spain to act to confront the demographic challenge in depopulation. Its region has five administrative divisions called provinces: Albacete, Ciudad Real, Cuenca, Guadalajara, and Toledo. The demographic vulnerability of Castilla-La Mancha through the indices of the European Commission in Region 2020 work [29] cites the interior territories of Cuenca and Guadalajara provinces as the most affected ones (Figure 1). Its law was approved on 12 May of 2021 with economic, social, and tax measures against depopulation and rural development. 

This demographic vulnerability is produced, amongst other aspects, because it is a region with a large territorial extension (with a surface of 79,463 km^2^, representing 15.7% of the national territory), that has had a historically low density of citizens (25.62 inhabitants per km^2^ in 2019). Indeed, it is characterized by an inconsistent territorial planning, where 919 municipalities are composed. A total of 80% (739) have at least 2000 inhabitants, which means that 15% of the regional population, and uniquely 36 municipalities, have 10,000 or more inhabitants, 56% of the total population. In Castilla-La Mancha, there have been different previous studies based on the different roles played by medium-sized cities [19,30] including functional areas [31] in the territory where influence together with expansion are exercised. These cities are priority objects of action, so as to maintain that community as more rural, yet with the selfsame options, services, and qualities to a distant that is sustainable in addition to reasonable.

The whys and wherefores confronting this challenge of territory, including demographic cohesion, are posed from a regeneration and integral urban development perspective in the medium-sized cities that supposed to, all planification ought to emanate from a diagnosis that allows to identify the main issues and necessities, as well as object population (relevance); it ought to establish clear and defined as well as analyzable (pertinence) objectives; and in the third place, the posed proceedings as ripostes to the detected problems and necessities ought to be adjusted to the achievements of goals (strategic coherence). Three aspects are proposed for Quality of Spatial Planning Design (CADIPAT) methodology by the Strategic Urban Agency to evaluate the quality of the design strategies of urban-territorial development [32].

### 3.2. The Province of Ciudad Real: Demographic Challenge

The province of Ciudad Real (495,761 inhabitants in 2019), where these two medium-sized cities are located (Ciudad Real and Puertollano), counts with 102 municipalities, 80% of which are rural and semi-rural (less than 10,000 inhabitants). This distribution of population makes these cities the principals and unifying hearts of the territory. The definition of the medium-sized cities of the province of Ciudad Real underscores its capital [33] (p. 148) and Puertollano [34] (pp. 203–204). 

**Figure 2:**
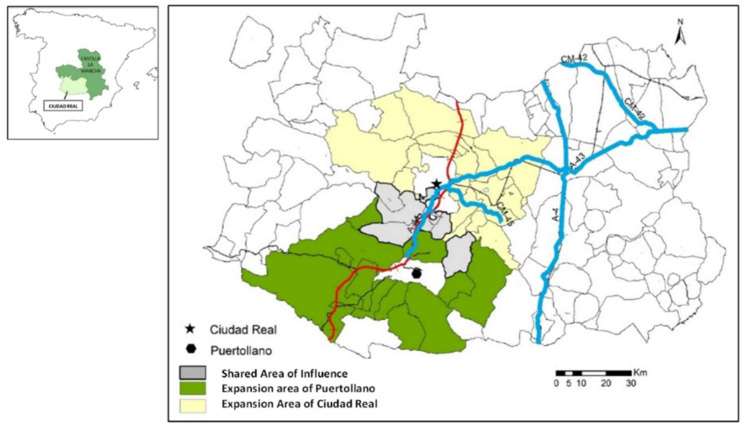
The Areas of Expansion of Ciudad Real and Puertollano, Source: [18] (p. 205).

For the delimitation of its respective areas of influence, previous works have been followed [35], determining a ratio of 30km surrounding the head [30]. The result has shown a crown of expansion of 25 municipalities for Ciudad Real^2^ and one of ten for Puertollano³ (Figure 2). 

With this paper, it was desired to relate the impact that these cities make in their crowns, bearing in mind the contribution of variables through the project “Depopulation and Depopulation in the Municipalities of the Province of Ciudad Real (D2CR)⁴”. For this goal, “growth variation range” is a variable that classifies all the nuclei of the province according to annual growth rate in the period of 20 years (1999–2019). It appears to be the most significant one, adding that there is a classification of seven ranges of all the municipalities of the province. According to this range of growth, the province is divided into seven ranges: the first three imply a positive growth in the last 20 years, while four values in the range represents stagnation, and values five, six, and seven adopt stagnation and therefore a demographic decline.

**Figure 3:**
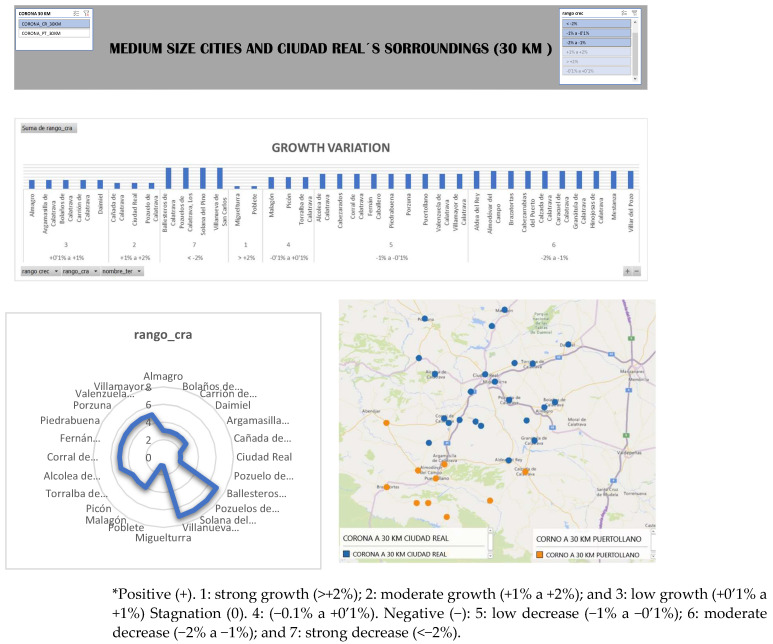
Growth Variation range in medium size cities of Ciudad Real province, Source: Compilation based on D2CR project. * This information under graphic Growth variation.

This classification allows to observe in the spider webs’ diagrams of the two cities the effect of Ciudad Real and its crown of expansion, where the majority of nuclei present values between 1–4 (Figure 3).

Our starting hypothesis employs a metaphor of the physical phenomenon of a drop of water that creates expansive concentric waves; as the same process happens through the urban process of medium-sized cities, our hypothesis chains that supporting dynamism of head medium-sized cities will have a beneficial effect on its urban expansion area, and therefore rural municipalities will benefit. 

### 3.3. Ciudad Real City and Its Urban Crown in Its Region

The study case has been carried out for the medium-sized city of Ciudad Real as a consequence of the impact that would generate in the province.

A first justification to work in the medium-sized city of Ciudad Real and its area of urban expansion, yet not in Puertollano, would be that according to the demographic size composed by 25 nuclei, which would postulate a population of 157,529, this represents 31.8% of the inhabitants of the province, in the face of 13.4% that Puertollano supposes (Table 1). 

Another reason is due to the role that Ciudad Real and its crown has had together with provincial joint with respect to demographic change in the last twenty years (1999–2019). It is where the two municipalities of the province that present the values of strong growth as well as moderate growth (range 1 and 2) belong practically to its totality to Ciudad Real’s crown. 

This shows that these are proactive nuclei so as to curb depopulation. Likewise, 50% of nuclei of the province have a stagnant position of growth (range 4), also belonging to its crown, and therefore they are nuclei that need to pay urgent attention before entering the demographic decline (Table 2). 

## 4. Aims, Materials, and Research Methods

Following this theoretical framework, the main aim of this paper is to propose a methodology to develop a project that impulses resilience strategies as well as revitalization in the local environments, and that will benefit the institutions that are involved in it, making a sustained study in the case of Ciudad Real state capital. In order to do so, the following will be envisioned:

− To establish the bases of the project named “We Propose!”, an educational project of citizen participation that aims at fostering student performance in the analysis and remedies proposal to the local dilemmas in the city, from an interdisciplinary perspective. Through classroom work, a field study of the students themselves is fostered. They interact with the environment from their way of living, contributing another perspective that is able to detect situations and propose adequate solutions to their needs.
− To elaborate a database emanating from presented projects about education and assessment of the local environment including urban regeneration. Valuing its potential together with deficiencies that allow to comprehend urban local inequalities, appointing areas of vulnerability as well as resilience strategies, utilizing their proposals as a new perspective of citizen participation.
− To analyse the different/innovative works provided to the city and typologies’ elaboration of drawbacks and proposals that, through the usage of statistical techniques, is multivariate. Thus, this geographic situation would permit to map the vulnerability as well as resilient strategies in Ciudad Real.
− To evaluate the transparency of erudition amongst the university, the world at large, as well as disparate institutions.
− To expose the successful cases of urban regeneration that were set out in the city, implying the public administration in its urban development. Yet, these were designed and proposed by the citizenry. Several instances that permit the opening of a debate are based on the reformulation of the dynamics of urbanization, including the governance of the medium-sized cities.

## 5. Characteristics of the Project

### 5.1. Genesis of We Propose! Project. A Citizen Participatory Urban Regeneration

*We Propose! Project* is an international project with the participation of more than 40 universities, 10,000, participants, and 6 countries. It started in Portugal in the academic year of 2011-2012 with Professor Sergio Claudino, professor of Geography at IGOT (University of Lisbon). 

In 2015, the Project was extended to Brazil, and in 2016 arrived in Spain, starting in Ciudad Real, the first Spanish city where this citizen participatory urban regeneration project was carried out, with the collaboration of University of Castilla-La Mancha (UCLM), department of Geography and Faculty of Education, and the City Council of Ciudad Real. In successive years, other cities from different areas of Spain, such as Valencia, Onteniente, Jávea, Córdoba or Alcázar de San Juan, and Miguelturra have joined the project. 

The incorporation of Spain in 2016, through the city of Ciudad Real, as an important step because it gave an Ibero-American character to the project. For this reason, the participation of Ciudad Real constituted a significant milestone of the project, and it commenced a new journey that was consolidated with the incorporation of another Spanish university, Valencia, in the following academic year 2017/2018

*Spanish´s We Propose! project* had two novelties with respect to its original project from Lisbon:

1. Institutional participation
2. The participation of the Primary Education centres

From the beginning, in Spain the channels of collaborations were established and needed for the transfer of knowledge amongst City Councils, Universities, including educational centres. This has led to two national recognitions, from the ministry of RETOS network Socially Sustainable Territories (2018), and from the association of Spanish Geographers (2021).

The project “We Propose!” in Spain pretends to promote the advancement of understanding within the challenges of the Horizon 2020: social innovations. It considers analysing the urban perspectives that the youth have for the city in which they live, generating these spaces of dialogue between the Public Administration and those who handle these spaces, the youth proposal together with the academic experts, bearing in mind the necessary training demanded by the city business network. 

### 5.2. “We Propose!” Project: Methodology

The methodology employed in this strategic design citizen participative urban renewal project denominated as “We Propose!” is the geographical analysis through “field trip” or investigation “in situ”. They enable the process of teaching-learning in the Geography of the classroom as well as outside of it. In the agreement, the management of the project generates “Project Based Learning (PBL)”, more specifically, in “Urban Problem Based Learning” (as this piece of work is orientated towards the solution of a challenge). Thus, it fosters a “Service Learning (SLA)” that gives an answer to the real necessities of the community by carrying out a utility service, including intellect with curriculum and urban regeneration of the city. 

### 5.3. “We Propose!” Project: As a Citizen Participation Strategy

*Service learning in urban regeneration* is worked on through the development of a different kind of class, in an emblematic space of the city and where the teachers are technicians from the City Council.

One of the most appealing informative sessions for the alumni is the one that is in a different classroom, and with other teachers, the technicians from the City Council. The attitude of the alumni is conventionally rather positive, mainly due to each alteration of routine change of the classroom awakening the interest. The informative session reinforces this motivation, in addition to providing civil and active education where students could know the functioning of the city including to whom it is addressed. In or out of the classroom, after receiving the pertinent explanations, the students have to select a theme or a drawback to study and constitute groups of work, with an active dynamic as well as collaborative.

*Exhibitions of the alumni at university and at the city council*

A relevant phase of the project is the presentations of the works, where the actuation of the teachers is to clarify as well as favouring that the exhibition of the students reflects on all the trajectory together with the obtained results. These exhibitions included all the finalist works at university and in the City Council in the plenary hall, and their authors are greeted by the mayor of the capital city.

### 5.4. “We Propose!” Project: Principles

**Figure 4:**
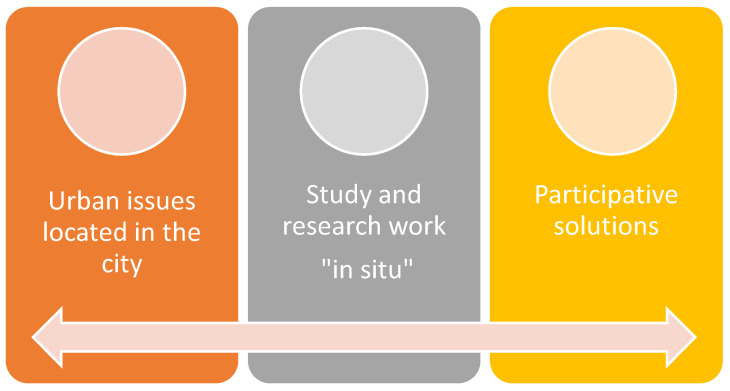
Principles of the Project “We Propose!” Source: [36].

The principles together with the methodology base of the We Propose! Project is supported by three pillars: (1) the identification of urban issues in the city, (2) the study and fieldwork or “in situ’’ investigation, and (3) the contribution of participative solutions (Figure 4). 

The “We Propose!” project dovetails problems as well as the local perception through the eyes of the alumni, generating conscience in the living environment. Thus, they would do so in a positive tone, as it no longer speaks about impediments. Instead, it touches on proposals as well as panacea. 

This educational orientation requires empowering the students’ capacities along with comprehension, adding the knowledge of specific geographic components [37], and that it has also been aggregated into three main objectives of this project:

1. To identify urban issues in the city, seeking to make students directly identify the natural constituent characters of the geographical space at a local scale. Comprehending the societal, cultural, and economic processes that have produced concrete shapes of occupation as well as utilization of geographical space.
2. The study and field work or investigation “in situ” enables them to handle statistical and traceable information as well as analysing and interpreting its distributions in the geographic space. It also permits them to know and utilize indirect knowledge techniques in all the scales—to relate behaviour or human performance standard with natural potential including the evaluation of its implications. Thus, they know how to integrate manifestations or local phenomena in natural, social, and economic scenarios at a larger scale.
3. To provide participative solutions of urban regeneration, the result of social awareness of the issue that fosters a knowledge of administrative and regulatory frameworks in force that condition the acts that have an incident in the territorial order; in addition, being able to work in interdisciplinary teams and utilizing language as well as contributions of other branches of knowledge.

Ultimately, this project seeks for personal involvement through understanding of close and tangible aspects together with those of distant situations that could provoke empathy in order to see the whole in a different perspective. 

### 5.5. “We Propose!” Project: Participants

The study subjects can be all schoolchildren from 5th and 6th grade of Primary School (10–12 years old and 1st and 2nd Secondary School (14–15 years old). In order to have a wide representation of society, students may come from schools of different social groups, from the public or private schools, and also from schools in marginalized areas. 

There are no admission criteria for participants; nonetheless, all those who apply should compromise to participate in all of the phases of the project, so as to include it in the general program of the center. These are reduced to three phases: (1) training of teachers; (2) training of students by technicians from the City Council; (3) expositions of the work at the University, City Council, and national and international meetings. 

### 5.6. National and International Meetings for Exchanging Knowledge

The national repercussions have been manifested in two national meetings. The first one was celebrated in Valencia in 2018, and the second one took place in Cordoba in 2018. 

Joining the Valencia project in 2018 made possible the realization of a meeting in this city in which the participating students of the different Spanish cities could present their works. The act was performed in Paraninfo of Universitat de Valencia and was presided over by the vice chancellor of education policy of this University, Ramón López. It was also led by the Councillors of Education of the City Councils of Ontinyent and from Ciudad Real, including the Director of the project in Lisbon, Sergio Claudino, as well as the coordinators Xosé Manuel Souto from Universidad de Valencia and María Ángeles Rodríguez Domenech from Universidad de Castilla-La Mancha. 

In February 2019, the assembly room of the Faculty of Education of Córdoba held the exhibitions of the selected works of this program that seeks for school pupils to work on the city issues as well as deal with proposed solutions. From Ciudad Real, multiple representatives from eight educational centers have participated. In this university, professors from the department of Geography endeavour to disclose the project amongst its university students together with making their first pilot experience with some Primary education centers of the city.

The *international repercussion* of this project leaves its distinctive mark in the “Ibero-American Forum on Education, Geography, and Society” (GEOFORO) including the celebration of the I Ibero-American Congress of the project We Propose! Citizenship, Sustainability, and Educational Innovation in September 2018.

There have been *three international meetings* celebrated in three different cities: Lisbon 2018, Ciudad Real 2019, and Estoril 2020. These meetings are intended to help strengthen the interrelations between students and teachers, universities, and politicians for each city that had participated in *We Propose! Project*. 

The *I International Meeting We Propose!* took place in Lisbon (Portugal) in 2018. The *II International Meeting We Propose!* took place in Ciudad Real (Spain) in 2019; participants included teachers as well as students from diverse Spanish locations (Ciudad Real, Alcázar de San Juan, Xábia, Ontinyent, Valencia, etc.) as well as Portuguese locations (Lisbon, Cascais, etc). The *III International Meeting We Propose!* took place in Estoril (Portugal) in March 2020, although it was originally planned in Cascais. However, it was not possible to celebrate it in this city due to the coronavirus (COVID-19).

### 5.7. Success Indicators: National Recognitions of We Propose! Project

"We propose!” The Spanish project has received two national acknowledgements from the Spanish Ministry and the Spanish Geography Association, both for its subject matter and for its educational innovation in the geographical work of the territory.

− In 2018, it was cataloged as a good practice in the “Sustainable Awareness Socially Responsible Territories Network (RETOS) of the Ministry of Spain”.
− In 2021, the originality of this initiative has been recognized officially. A few weeks ago, the Spanish Geography Association awarded this project with the I Award to the “Educational Innovation in Geography”, spotlighting both the methodology applied as well as its thematic opportunity.

## 6. Result: We Propose! Project in Ciudad Real 

### 6.1. Participation in We Propose! Project in Ciudad Real

**Figure 5:**
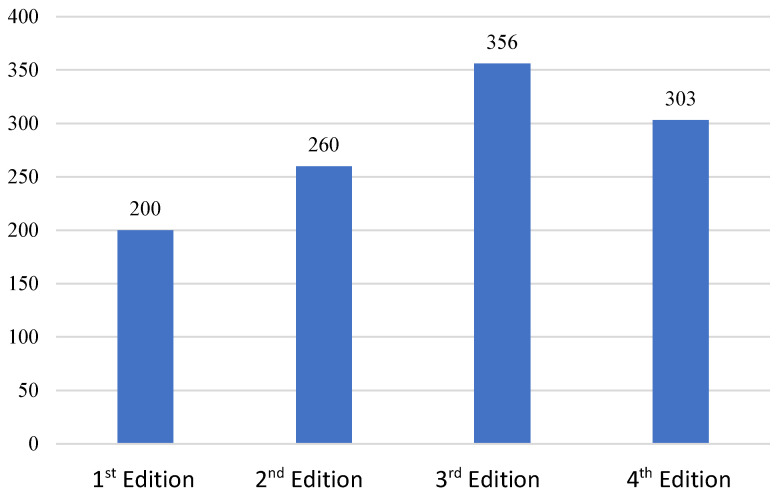
Evolution of participants per edition, Source: Own elaboration.

The first edition (2016–2017) had a total of 200 participants from Ciudad Real capital. In the second year, this participation heightened to 260 participants from Ciudad Real as well. The involvement of the third edition (2018–2019) ascended notably to 356 due to the amplification of the participating cities, being the first edition where it counted two participating cities (Ciudad Real, Alcázar de San Juan). In the fourth edition (2019–2020), the numbers of participants stabilized to 303, counting with three participatory municipalities (Ciudad Real, Alcázar de San Juan, and Miguelturra) (Table 3 and Figure 5).

**Figure 6:**
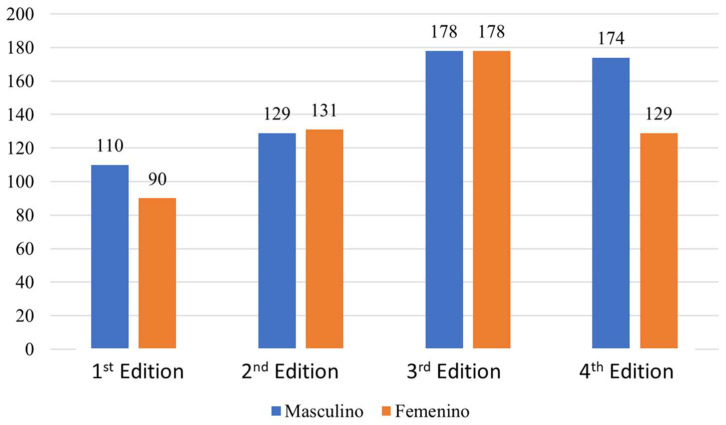
Distribution per sexes of the different participants, Source: Own elaboration.

With respect to the type of education of the participating students, it could be observed that the following graphic as well as the Primary alumni suffered a growth from the third edition. Nonetheless, the Secondary alumni maintained a more constant line of participants. In reference to the gender of the participants, it could be observed that in the first and fourth edition, the number of female students was lower than male students. In the second and third editions, the ciphers were fairly even. In total, 591 male students together with 528 female students participated in these four editions (Figure 6).

The distributions per sexes in the different participants is represented. However, age was not considered as it was not one of the demographic variables in mind. Age bracket in the course was uniquely registered in this project. They are students from Primary School (10–12 years old) and students from Secondary School (between 13 and 16 years old). 

**Figure 7:**
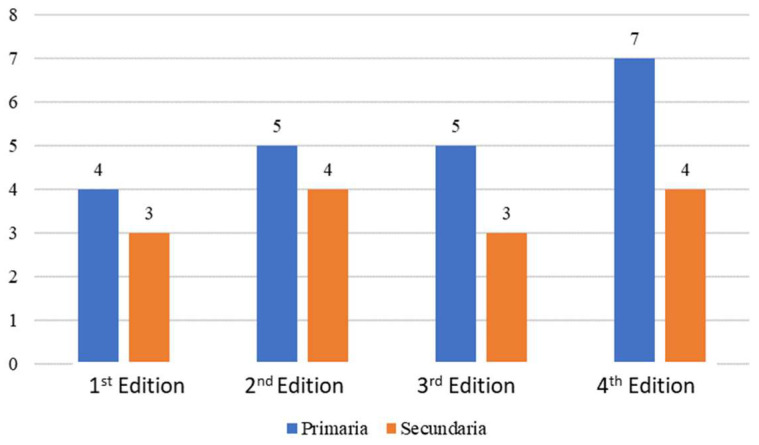
Primary and Secondary participants centres per Edition, Source: Own elaboration.

In relation to the *participatory centres*, it could be affirmed that there has been an increment. In this way, the total number of participatory centres in these years has been 35 (21 Primary and 14 Secondary School). Notwithstanding, many of these centers have repeated the same experience while others have decided to live it once. There are three unique centers that took part in the four editions: Pio XII (Primary School), San José, and Hernán Pérez del Pulgar for Secondary School (Figure 7). 

If the participating centres per edition were disaggregated, the following could be found:

**1st edition:** From **Ciudad Real**: Primary School (Carlos Vázquez, Cristóbal Colón, Ferroviario, and Pío XII) and Secondary School (Nuestra Señora del Prado, San José, and Hernán Pérez del Pulgar).

**2ndedition:** From **Ciudad Real**: Primary School (Alcalde José Cruz Prado, Cristóbal Colón, Ferroviario, Miguel de Cervantes, Pío XII), and Secondary School (San José, Hernán Pérez del Pulgar, Maestro Juan de Ávila, and Torreón del Alcázar).

**3rd edition:** From **Ciudad Real**: Primary School (Miguel de Cervantes, Alcalde José Cruz Prado, Salesianos Hermanos Garate, and Pío XII) and Secondary School (Hernán Pérez del Pulgar, San José, and Torreón del Alcázar). De **Alcázar de San Juan**: Primary School (Pablo Ruiz Picasso) and Secondary School (Miguel de Cervantes and Juan Bosco).

**4th edition:** From **Miguelturra**: Primary School (Santísimo Cristo de la Misericordia and Maria Elena Maseras), Nuestra Señora de la Merced (Primary and Secondary); **Ciudad Real**: Primary School (Jorge Manrique, Pio XII), and Secondary School (San José and Hernán Pérez del Pulgar); **Alcázar de San Juan:** Primary School (Pablo Ruiz Picasso) and Secondary School (Miguel de Cervantes and Juan Bosco).

On the other hand, the participation of the teachers has increased with respect to the first year. In general terms, one teacher per centre participates in the centres of Secondary education, except for Hernán Pérez del Pulgar (Secondary School), which takes part with two parallel teachers. In the cases of Primary education, as the alumni need more attention due to being minors, it counts with two teachers per class of parallel classes.

### 6.2. Evolution of the Content of the Students´ Work Presented at We Propose! Project

In relation to the worked themes for the participation of the different editions of the project, it could be observed how they have been evolving not only regarding innovation of proposals but also the perfect methodology of the project. 

Therefore, in the **1st edition** there is a tendency for conservation including restoration of patrimony, both in Primary and Secondary seeking alternatives of leisure for their age emanating from the restoration of disused buildings.

In the **2nd edition,** students‘ work maintains the preoccupation for the restoration of the patrimony of the cities both in Primary as well as Secondary Education. Nevertheless, there is a Primary tendency for the amelioration in the urban neighbourhoods in relation to the security, both personal together with sidewalks, urban furniture, and problems of coexistence. In Secondary School, there is the incorporation of technologies of information and communication with the creation of apps for the mobile phone that facilitates mobility. The environment through the amelioration along with innovation of gardens in the city make a powerful innovative entrance in the type of presented projects, as the contribution of viable solutions such as parking or main transportation in the city. 

In the **3rd edition,** the variety of proposals is widened, although it prevails themes related to sustainable mobility such as: the usage of bicycles including the rehabilitation of buildings for social and cultural usage. In Secondary education, there is maintenance for the preoccupation of environmental aspects (rainfed gardens, vertical gardens, ecopoints).

Ultimately, in the **4th edition** the incorporation of new cities provokes that the issues of concern for both stages, Primary and Secondary, are vastly varied, although the aim of the project predominates in all cases. There are proposals of improvement for sustainability, innovation, as well as citizen coexistence. This is why there are projects related to the rehabilitation of spaces, care for the environment, spaces for the disabled, inclusion of leisure elements…

It could be affirmed that the students’ proposed work presents an evolution related to the betterment of the architectonic elements of the cities, not only for coexistence, the adaptation of impairment, the enhancement in the environment, aesthetic proposals that give a particular beauty to the city, for instance vertical gardens. A wide variety has been forged, a fully innovative and multidisciplinary variety that consolidates the aim of the project. 

**Figure 8:**
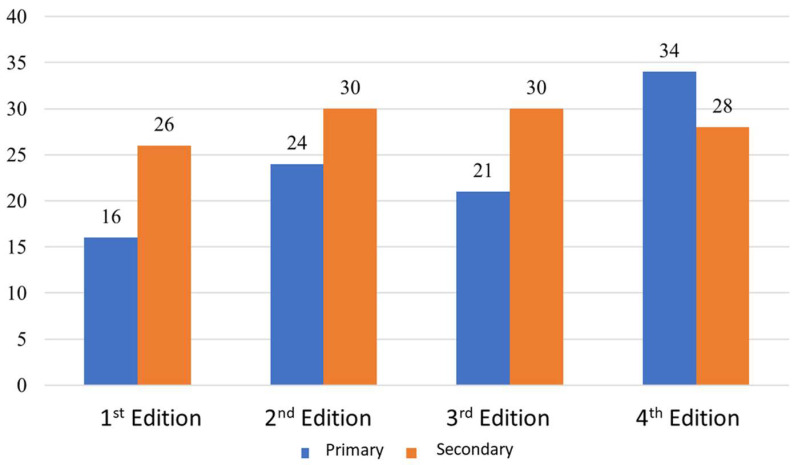
Evolution of students’ work presented per edition, Source: Own elaboration.

With respect to the numbers of projects presented, it could be regarded in Figure 8 that there is a small fluctuation in the third edition of Primary education, which is insignificant, while in Secondary it is stable, demonstrating a minor variation concerning numbers (Figure 8). 

### 6.3. Contributions of Participatory Urban Regeneration Students‘ Work and Their Relationship with Sustainable Development Goals

The proposals work with various themes related to the patrimony, environment, mobility, social, leisure, and new technologies. It could also be appreciated how the majority of the proposals underscores the theme about patrimony, environment, mobility together with leisure. Additionally, with these proposals, 17 Sustainable Development Goals (ODS) will be exploited: 9. Industry, innovation, and infrastructures; 11. Cities and sustainable communities; 13. Action for the climate; 15. Life of terrestrial ecosystems, and; 16. Peace, justice, and solid institutions. 

After reading the proposals, it could be observed that the most prominent concerns were contamination, environment, rehabilitation of buildings, accessibility, and bad conditions as well as services that the poorest neighbourhoods of Ciudad Real have, points in the city, basic services coupled with arrangement of infrastructures in the least developed neighborhoods of Ciudad Real.

It could be perceived that there have been 4 or 5 proposals in each center, creating a total of 18 proposals. Yet, ascribed to the basis of the contest, two of each center could have been selected exclusively, and this is why the idea could not be transformed into an image. Amongst them, one could highlight heritage themes, environment mobility, SDGs 11, 13, and 15, contamination, bad services, and conditions of the least developed neighborhoods of Ciudad Real, environment, and poor accessibility in certain points, which are mainly the most discussed matters. 

Notwithstanding, among the selected proposals, 13 in total have focused more on the patrimony, leisure, SDGs 9 and 16, the rehabilitation of buildings’ themes as a new place for leisure, and security (including vandalism) as the most concerning matters. 

The themes, sustainable development goals, and methodology carried out in the developed projects of the proposals are the following:

− The proposals of the selected sheets are commonly about themes related to the cultural patrimony of the city as it exits a preoccupation for recovery, promotion, and comprehension of cultural patrimony of the city in which there already exists a preoccupation for recovery, promotion, and knowledge of cultural patrimony of the city. Additionally, many of the proposals come into play in the rehabilitation of uninhabited buildings, or they could be reformed for social purposes such as poverty reduction or the integration of different social groups. Each of these cards pursue goals in relation to the EU’s sustainable development objectives that are related to quality of education, industry, innovation, and infrastructure, as well as the reduction of inequalities, zero poverty, health, and well-being. Consequently, to be able to ascertain the facts of the matter that the city has detected besides the preoccupations of the citizens, the alumni have utilized different methodologies such as: field trips, surveys, interviews, and research on journalistic backgrounds.
− Bearing in mind the theme, sustainable development goals, and the methodology of the non-selected sheets, it could be perceived that the most concerning matters to the alumni are related to the rehabilitation of unused buildings for social purposes (people with disabilities, reducing inequalities, enlarging cultural heritage, tourism, and leisure). It could also be observed that there is a preoccupation with health and environment, including measures such as the creation of sustainable spaces such as urban gardens, solutions to save water, recycling, and pollution. As regards, each of the proposals as a whole meet the overall goals of sustainable development, as all of them stage in the attainment of the sustainable expansion favorable for our city. Furthermore, the alumni have considered through the methodology implemented the realization of the proposals to the citizens of our province through the realization of questionnaires, together with direct interviews, carrying out a project of direct observation, inquiring into the issues, and taking the examples of other projects that have succeeded in other cities.

### 6.4. Success Stories: Participatory Urban Regeneration Students’ Work That Have Become a Reality

“Success students’ work” have been considered those proposals that have been put forward in the cities— in other words, those that were contemplated and brought forward by the City Council. This does not signify that the remaining were not deemed as valid or prosperous for the design of the thematic. Instead, they have not been made yet. 

Within more than 180 proposals, certain presented projects by the students have been executed and implemented in the city with institutional support. In the case of Ciudad Real, two projects have been set up: “Everyone’s Responsibility: Pet Spreading” by Pio XII Primary School in 2018 and “Vertical Garden” by Hernán Pérez del Pulgar Secondary School in 2020. Thus, the City Council of Miguelturra has not run any project. Yet, they has announced that they intend to implement the project about graffiti “Carnival Walls” for 2022. 

## 7. Evaluation 

### 7.1. Evaluation of Quality of Spatial Planning Design of Students ‘Work

The evaluation of the quality of the design of urban-territorial development strategies of the projects has been carried out in the papers. Yet, the analysis of an urban regeneration project proposed by the school Pío XII for a marginal area of the city, conditioning a green area next to the school, will be shown as a sample. 

**Figure 9:**
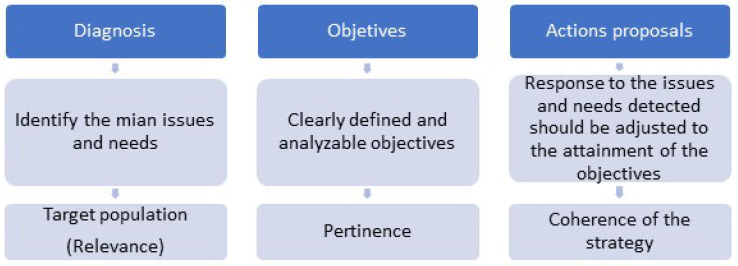
Evaluation of the quality of the design of urban-territorial development strategies, Source: Own elaboration based on CADIPAT PROJECT [38].

In order to evaluate the strategy of participative integral urban regeneration, the following outline will be followed (Figure 9):

### 7.2. Evaluation of Students‘ Work “Everyone’s Responsibility: Pet Spreading” by Pio XII Primary School 

This project was run by the students of 6th Primary (12 years old) from a school of difficult performance in the city Pío XII, situated in one of the most vulnerable slums of the city, where the majority are recognized gypsy ethnicity, and they are poorly integrated in the city (Table 4, Table 5 and Table 6).

### 7.3. Social Impact: Network Visibility and Scientific Dissemination

Social means of communication have a tremendous impact on public opinion. There should be a differentiation between two types of means of communication, known as old media, which includes TV, radio, newspaper, etc., and the new media or social media. The latter comprises basically those means based on the internet, such as Twitter and Facebook, which permit the interaction, conformation of networks, and groups including content exchange. The traditional means of communication already had a massive impact on the individuals; be that as it may, new media has become much more efficient, through mechanisms that have replaced direct social interaction through virtual social media [39].

Throughout these four editions, the apparitions of these means of communications have increased. Since the first edition of the project, it gained invaluable support from the communication cabinet of the City Council of Ciudad Real, which made numerous pieces of press releases, photographs and reports about the evolution of the project. Additionally, each of the alumni’s exhibitions were retransmitted live through *Facebook*, offering a broad visibility of it. 

The creation of the website https://www.nosotrosproponemos.com, (accessed on 1 January 2022) has boosted the project to other cities of Spain, offering information about the basis of the piece of work, pieces of news, papers, congresses, etc. However, without a doubt it has been the Blogs of diffusion of Geography http://blogdegeografiadejuan.blogspot.com/p/proyecto-nosotros-proponemos.html (accessed on 31 January 2022) that has provided a wider diffusion. 

**Figure 10:**
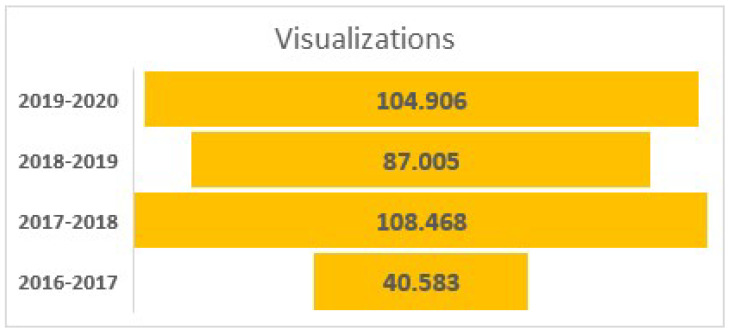
Visualizations of We Propose! Project, Source: Geography blog by Juan Martín Martin.

Thus, out of 23,509 visits to the official website, the visits to the Geography Blog of 40 presentations submitted to the blog has been seen 340,962 times, which indicates a very large diffusion coupled with consultations of results of the project, not only in Spain but also in other countries (Figure 10).

## 8. Discussion 

The implementation of certain presented projects is a guarantee of its good functioning, including the level of satisfaction of all the implicated agents, even though their proposals have not been brought forward. Ultimately, the expansion of the program to other cities has been contemplated. This is an indication of its appropriateness and relevance.

In relation to the planned goals, it could be concluded that with respect to the ***design of the urban regeneration project:***

− The implication of the City Council to formulate some of the presented proposals is an indicator of the high level of viability of the assignment done by the citizens’ participation.
− The geographic methodology implemented in the design of the projects guarantees an analysis, diagnosis, and suggestions that show the quality of the method.
− The scientific publications are of the program.
− With respect to the presented projects:
− The membership to a city national, including international network “We Propose! Project”, permits to amplify and strengthen ties with other cities and, therefore, with other perceptions together with ripostes, global-local, entirely enriching to all.
− The themes addressed have a tremendous repercussion in the Sustainable Development Goals.
− In the current society, there is a considerable interest in the projects of local urban renewal, as shown by the large diffusion that includes the number of visits received in the communication channels.

Lastly, being conceivably one of the most relevant aspects regarding ***transparency of knowledge to society***:

− The exhibition of the projects at the University and City Council is one of the most valued aspects of the project, as it has created a new channel of active citizens’ involvement. It creates a culture of dialogue equally and amongst multiple administrations.
− It brings together the technical services of the City Council, making both teachers as well as counsellors for student’s ideas. This would let them create honest citizens implicated in their cities.
− The creation of the channels of communication among teachers and students of different educational levels (Primary, Secondary, and university education). They are at dissimilar socio-economic levels, which boosts a more inclusive and tolerant society.

A future line of work and investigation would be the replication of this program in other nuclei based on the level of success obtained in Ciudad Real. The bet would consist of the nuclei of the crown of urban expansion of Ciudad Real, replicating the project in its respective localities. This would contribute and foster its youngest inhabitants to feel actively more integrated in the resolutions of the dilemmas of their localities. Consequently, this would create an augmentation of the entrepreneur spirit and belongness, supposing a revitalization from the roots by reducing socio-demographic vulnerability.

## 9. Conclusions

The proposal of urban renewal Next Generation through the design together with the evaluation of the project We Propose! in Ciudad Real has been a positive experience and therefore replicable in other cities of minor entities. As pointed out by the New Urban Agenda 2030 in its prologue: “there are strong linkages between good urbanisation and job creation, livelihood opportunities, and improved quality of life, which should be included in all urban renewal policies and strategies”.

Amongst the principal conclusions of this type of participative initiatives, it could be spotlighted that it is possible to draw plans for more urban planning different from what has been done before. It was not only a theoretical proposal, but also something that could be turned into reality, being more favorable for medium-sized and small cities due to its magnitude. 

It is acknowledged that these instances open up a debate about the reformulation of the dynamics of urbanism, including governance of the medium-sized cities. The revitalization of spaces could be mapped out from the basis of citizenship.

## Figures and Tables

**Table 1:** Typology by inhabitants of the province through their medium-sized cities. Source: Compilation based on *ID2CR project: Depopulation and depopulation in Ciudad Real*.

Typology by Inhabitants	Range of Cities	Ciudad Real		Ciudad Real and Crown			Puertollano and Crown
		Inhabitants	No. Nuclei	Inhabitants	No. Nuclei	% Inhabitants 2019	% Inhabitants 2019
1: <200	Rather small rural	481	4	311	3	0.2	0.0
2: 201–1000	Small Rural	22,882	41	3439	6	2.2	3.9
3: 1001–2000	Medium Rural	26,029	20	5190	4	3.3	1.5
4: 2001–5000	Intermediate Small	46,015	14	2165	6	12.8	5.6
5: 5001–10,000	Intermediate Big	77,787	11	8447	2	5.4	18.0
6: 10001–20,000	Small Urban	104,260	7	45,231	3	28.7	0.0
7: 20,001–50,000	Medium Urban	143,561	4	0	0	0.0	70.9
8: 50,001–100,000	Big Urban	74,746	1	74,746	1	47.4	0.0
	Total	495,761	102	157,529	25	31.8	13.4

**Table 2:** Typology of *Growth Rate* in the province with respect to Ciudad Real and surroundings, Source: Compilation based on *ID2CR project: Depopulation and depopulation in Ciudad Real*.

Change of Growth Range (1999–2019)			Ciudad Real Province			Ciudad Real and Surroundings	
		Group	Population 2019	No. Municipalities	%	No. Municipalities	% Regarding the Province
**1: > +2%**	Strong Growth	+	17,994	2	2.0	2	100.0
**2: +1% to +2%**	Moderate Growth	+	114,274	4	3.9	3	75.0
**3: +0.1% to +1%**	Weak Growth	+	192,508	14	13.7	4	28.6
**4: −0’1% a +0.1%**	Stagnation	0	24,916	6	5.9	3	50.0
**5: −1% to −0.1%**	Weak Decrease	-	93,193	24	23.5	7	29.2
**6: −2% to −1%**	Moderate Decrease	-	49,711	44	43.1	4	9.1
**7: < −2%**	Strong Decrease	-	3165	8	7.8	2	25.0
	TOTAL		495,761	102	100	25	24.5

**Table 3:** Participation in the project with Editions and Variables.

	1st Edition of the Project	2nd Edition of the Project	3rd Edition of the Project	4th Edition of the Project
**Participants (students)**	200	260	356	303
**Primary and Secondary education female and male Students**	103 Primary and 97 Secondary	107 Primary and 153 Secondary	233 Primary and 123 Secondary	198 Primary and 105 Secondary
**Gender**	110 men and 90 women	129 men and 131 women	178 men and 178 women	174 men and 129 women
**Participating centres**	7	9	8	11
**Primary and Secondary centres**	4 Primary centres and 3 Secondary	5 Primary centres and 4 Secondary	5 Primary and 3 Secondary	6 Primary centres and 5 Secondary
**Private centres**	2	1	2	2
**Primary and Secondary teachers**	11 (6 Primary and 5 Secondary)	15 (9 Primary and 6 Secondary)	16 (8 Primary and 8 Secondary)	18 (10 Primary and 8 Secondary)
**Participating cities**	1 Ciudad Real	1 Ciudad Real	3, Ciudad Real, Alcázar de San Juan, and Jávea	3, Ciudad Real, Miguelturra, and Alcázar de San Juan
**Presented works**	16 Primary and 26 Secondary	24 Primary and 30 Secondary	21 Primary and 30 Secondary	62 works. 34 Primary and 28 Secondary
**Press appearances**	10	12	14	18

Source: Own elaboration.

**Table 4:** Diagnosis of the students’ work “Everyone’s Responsibility: Pet Spreading” by Pio XII Primary School.

**LOCALIZATION** **NEIGHBOURHOOD** **STATUS** **Methodology: Project-Based Learning**	The Neighbourhood Is Composed of Houses as Well as Huts, Where the Majority of Their Neighbours Are Recognized Gypsy Ethnicity. The Trash, the Lack of Care of Gardens, Flooded Streets, Violence, and Even Rats Could Be Seen Daily.
**CITIZEN PERCEPTION** Methodology: Enquiries	- Over 95% of the total of the respondents voiced the same. Most of them have lived in the neighbourhood since they were born and are not keen on living there. If they could, they would move out to a district with “PAYOS”. - To others (90%), they enjoyed their neighbourhood. However, they admitted that the school needed to be amended, although the option of opening up a community center was perceived positively and, therefore, they would be willing to participate in the liveliness. - To set off from this marginal situation is arduous, and even more so when boys drop out of schooling too early, without even having enough formation to look for a decent job. - It was utterly easy to make the questionnaire as all the neighbours approached and facilitated the study (they were pleased to see how the offsprings did the activities in the district). - When planning the enquiry in other districts, the reception was not the same. Thus, there were fewer inhabitants in the streets, and most of them ran away from us.
**FRAMING ISSUE** Methodology: field work	- To fix streets, cleaning in general, new paving, etc. Once the school was left behind, information about everything perceived was collected. - It was discerned that the entrance of the school presented an unfavourable image. It was full of dog excrement.
**EDUCATIONAL INSTITUTION SCHOOL** (adult’s perception of the neighbourhood))	- The school plays a key role that attempts to ameliorate this situation. Opening up a centre to ALL could transform the future of this neighbourhood. Literary workshops for the elderly, educational workshops for the young adults, musical workshops, cooking workshops, sports, literary gatherings (there are plenty of books in the educational institution), etc. - It would be great to take advantage of the liveliness, and as a consequence, each of the neighbours of the multiple districts would utilize this space to find pleasure in it. Thus, they could nurture inclusion of infants and families.

Source: Own elaboration.

**Table 5:** Goals of the students’ work “Everyone’s Responsibility: Pet Spreading” by Pio XII Primary School.

Goals	Proposed Actions
**Green Areas “Healthy Gardens”**	- The “garden” that is in the entrance would be converted into a precious green area with even a children’s playground with a special area for pets.
**Educational building with “Graffiti’s Educational”**	- To make improvements in the aspect of the facades. The ugly paintings would be converted into powerful messages to those who walk down the street.
**Sustainable awareness “Vegetable Garden** **escolar”**	- Reutilization of an owned abandoned sandbox (full of grass as well as garbage thrown from the street) to adapt to a “Vegetable Garden Escolar”/sustainable consciousness; in addition to learning from them, families, amongst all, could take part in its maintenance.

Source: Own elaboration.

**Table 6:** Results of the students’ work “Everyone’s Responsibility: Pet Spreading” by Pio XII Primary School.

Typology	Results	Evaluation
**Urban Regeneration**	- The proposed actions were carried out in 2018 (“Garden”, “school front”, and “vegetable garden”) with budgetary involvement of the City Council.	- In 2021, there is maintenance and respect of all the citizens of the neighbourhood.
**Entrepreneurial spirit**	- Students and families looked at the answer to augment the usage of educational centres including other activities to other groups in the district.	- The participation in and usage of the spaces have increased and involved different agents.
**Inclusion**	- The participation as equals in the contest as other infants of other centers of the city has been positive, for both the alumni and teachers.	- The integration and no inclusion of the participation of all types of centers: public, privates, and difficult performance is valued quite positively for all the implicated agents.
**Socio-Economic Regeneration**	- The enhancement of the environment of the educational center has favoured the creation of local businesses	- Economic activation of the area.

Source: Own elaboration.

## Data Availability

Not applicable.

## References

[1] Brenner N., Christian Schmid C. (2015). Towards a new epistemology of the urban?. City.

[2] Lois R.C., Piñeira M.J., Vives S. (2016). El proceso urbanizador en España (1990–2014): Una interpretación desde la geografía y la teoría de los circuitos de capital. Scr. Nova.

[3] Gil F., Bayona J. (2012). La dinámica urbana en España: Evolución y tipología. Pap. De Geogr..

[4] Olazabal E., Bellet C. (2019). De la ciudad compacta a la ciudad extensa. Procesos de urbanización recientes en áreas urbanas españolas articuladas por ciudades medias. An. De Geogr. De La Univ. Complut..

[5] Sposito C., Bellet C. (2009). Las Ciudades Medias o Intermedias en un Mundo Globalizado.

[6] Bellet C., Llop J.M. (1999). Ciudades Intermedias y Urbanización Mundial.

[7] Martínez Navarro J.M., García González J.A., Y Escudero Gómez L.A. (2020). Las ciudades medias de España y sus coronas en el siglo XXI (2000–2017): Dinámica demográfica y desarrollo inmobiliario. Urbe.

[8] Górgolas Martín P. (2020). Regeneración y Planeamiento para Ciudades Sostenibles. Experiencias en América, Marruecos y España.

[9] Górgolas Martín P. (2019). Del “urbanismo expansivo” al “urbanismo regenerativo”: Directrices y recomendaciones para reconducir la herencia territorial de la década prodigiosa del urbanismo español (1997–2007). Aplicación al caso de estudio del litoral andaluz. Ciudad. Y Territ. Estud. Territ..

[10] (2011). Cities of Tomorrow: Challenges, Visions, Ways Forward. European Union. http://ec.europa.eu/regional_policy/sources/docgener/studies/pdf/citiesoftomorrow/citiesoftomorrow_final.pdf.

[11] Espino Hidalgo B., Navas Carrillo D. (2018). Planeamiento estratégico local y evaluación del desarrollo urbano sostenible integrado en ciudades medias. GAPP.

[12] REDS (2020). Los ODS en 100 Ciudades Españolas.

[13] Fernández D., Corbelle E. (2017). Cambios en los usos de suelo en la Península Ibérica: Un meta-análisis para el período 1985–2015. Biblio3W.

[14] Bellet C. (2020). Municipal planning policies in Spain: 40 years of democratic city councils (1979–2019). Boletín De La Asoc. De Geogr. Española.

[15] Cebrián F. (2020). Los procesos de transformación de las ciudades medias. De la ciudad compacta a la metástasis territorial en España. Dinámicas de Urbanización en Ciudades Medias Interiores ¿Hacia un Urbanismo más Urbano? Cebrián, F., Ed..

[16] Indovina F., Martín Ramos A. (2004). La ciudad difusa. Lo Urbano en 20 Autores Contemporáneos.

[17] Del Valle C., Almoguera P. (2020). Envejecimiento demográfico y (des)población en las ciudades medias. Cuad. Geográficos.

[18] Bellet Sanfeliu C., Andrés López G. (2021). Urbanización, crecimiento y expectativas del planeamiento urbanístico en las áreas urbanas intermedias españolas (1981–2018). Investig. Geográficas.

[19] Mendez R., Michelini J.J., Romeiro P., Sánchez Moral S. (2006). Ciudades intermedias y desarrollo territorial en Castilla-La Mancha. Xeográfica.

[20] Bellet C. (2017). Proyectos y grandes operaciones urbanas. Naturaleza, Territorio y Ciudad en un Mundo Global.

[21] Cebrián Abellán F., Sánchez Ondoño I. (2019). Urban Sprawl in Inner Medium-Sized Cities: The Behaviour in Some Spanish Cases Since the Beginning of the 21st Century. Urban Sci..

[22] Cebrián F., Panadero M. (2013). Ciudades Medias. Formas de Expansión Urbana.

[23] Cañizares M.C., Rodríguez-Domenech M.A. (2020). “Ciudades fantasma” en el entorno del Área Metropolitana de Madrid (España). Un análisis de la Región de Castilla-La Mancha. EURE.

[24] United Nations Sustainable Development Summit 2015. https://sustainabledevelopment.un.org/post2015/summit.

[25] United Nations (2016). Pact of Amsterdam. https://ec.europa.eu/regional_policy/en/information/publications/decisions/2016/pact-of-amsterdam-establishing-the-urban-agenda-for-the-eu.

[26] Rubio-Huertas E., Ureña-Francés J.M. (2021). Evaluación de la efectividad en la regeneración urbana de nuestras ciudades. Lecciones aprendidas. Ciudad. Y Territ. Estud. Territ..

[27] Moulaert F., Parra C., Swyngedow E. (2014). Ciudades, barrios y gobernanza multiescalar en la Europa urbana. EURE.

[28] Navarro C. (2015). Mejorar la Ciudad Transformando Sus Barrios. Regeneración Urbana en Andalucía (1990–2015).

[29] Rodriguez-Domenech M.A. (2016). Vulnerabilidad demográfica en las regiones europeas Nuts-2. El caso de Castilla la Mancha. Rev. Pap. De Población.

[30] Cebrián Abellán F. (2007). Ciudades con límites y ciudades sin límites. Manifestaciones de la ciudad difusa en Castilla-La Mancha. Boletín De La Asoc. De Geogarafía Española.

[31] Pillet F., Cañizares M.C., Ruiz A.R., Martínez Sánchez-Mateos H., Plaza J.J., Santos Santos J.F. (2018). Dinámicas demográficas y su relación con la cohesión territorial en las áreas funcionales urbanas de Castilla-La Mancha (España). Boletín De La Asoc. De Geogarafía Española.

[32] Guerrero-Mayo M.J. (2020). Evaluación de la Calidad del Diseño de Estrategias de Desarrollo Urbano-Territorial. Manual Para su Análisis Mediante la Aplicación CADIPAT.

[33] Rodríguez-Domenech M.A. (2012). Nueva Realidad Urbana y Territorial de Ciudad Real (1980–2010).

[34] Ramirez Lopez L.J., Grijalba Castro A.I. (2021). Sustainability and Resilience in Smart City Planning: A Review. Sustainability.

[35] Cañizares M.C., Rodríguez-Domemech M.A., Castanyer Vivas M., Vicente Rufí J., Uceda J.F.T.y.J.M. (2017). Castilla-La Mancha y sus nuevos escenarios urbanos: Las ciudades fantasma. Nuevos Escenarios Urbanos: Nuevos Conflictos y Nuevas Políticas.

[36] Rodríguez-Domenech M.A., Claudino S. (2018). ¡Nosotros Proponemos!: Ciudadanía, Sostenibilidad e Innovación Geográfica Ante los Desafíos Educativos de la Sociedad.

[37] Zoido Naranjo F. (1998). Nuevas fronteras de los contenidos geográficos. Íber.

[38] Guerrero-Mayo M.J., Rodríguez-García M.J., En Navarro C.J., Rodríguez-García M.J., Guerrero-Mayo M.J. (2020). La planificación del desarrollo urbano integral: Calidad del diseño y aprendizaje. Lógica e Impactos de la Estrategia Integral en Políticas Urbanas.

[39] Terán O., Aguilar J.L. (2018). Modelo del proceso de influencia de los medios de comunicación social en la opinión pública. Educere.

